# A Pilot Event-Related Potentials Study on Mechanisms Underlying a tDCS-Enhanced Food-Specific Response Inhibition Task for Patients With Binge Eating Disorder

**DOI:** 10.3389/fpsyg.2021.721672

**Published:** 2021-10-12

**Authors:** Başak İnce, Sebastian M. Max, Christian Plewnia, Elisabeth J. Leehr, Stephan Zipfel, Katrin Elisabeth Giel, Kathrin Schag

**Affiliations:** ^1^Department of Psychology, Haliç University, Istanbul, Turkey; ^2^Department of Psychiatry and Psychotherapy, Neurophysiology & Interventional Neuropsychiatry, University of Tübingen, Tübingen, Germany; ^3^Institute for Translational Psychiatry, University of Münster, Münster, Germany; ^4^Department of Psychosomatic Medicine & Psychotherapy, University Hospital Tübingen, Tübingen, Germany; ^5^Centre of Excellence for Eating Disorders (KOMET), University Hospital Tübingen, Tübingen, Germany

**Keywords:** binge eating disorder, response inhibition, impulsivity, antisaccade, cognitive control, event-related potentials

## Abstract

Behavioural studies demonstrate alterations in cognitive functioning, particularly impaired response inhibition and increased attentional bias towards food in binge eating disorder (BED). This pilot study aimed to investigate the neurophysiological processing of a food-specific inhibition training combined with anodal transcranial direct current stimulation (tDCS) of the right dorsolateral prefrontal cortex (DLPFC) in 16 patients with BED (mean age = 38.6, mean BMI = 33.7 kg/m^2^). Patients performed a food-specific antisaccade task at baseline (T0) and in a cross-over design with verum vs. sham stimulation at T1 and T2. We investigated (i) event-related potentials (ERPs; N2, ERN and P3 amplitudes) while executing the task at baseline, (ii) whether baseline ERPs would predict task performance at T1 and T2 and (iii) associations between ERPs, eating disorder pathology and impulsivity at baseline. The mean amplitude of N2 was less pronounced in erroneous saccades (ES) than correct saccades (CS), whereas ERN and P3 mean amplitudes were more pronounced in ES. Moreover, the P3 mean amplitude of ES predicted the percentage of ES at both follow up-measurements irrespective of the applied stimulation (sham vs. verum). N2 in trials with correct saccades were negatively correlated with nonplanning trait impulsivity, while P3 in erroneous antisaccade trials was negatively correlated with food-related impulsivity. Overall, the findings of reduced ERN, enhanced P3 and N2 amplitude might be interpreted as difficulties in response inhibition towards food in individuals with BED. In particular, P3 predicts task outcome at follow-up and might represent a potential marker for inhibitory control processes.

## Introduction

As the most recent eating disorder (ED) diagnostic category in the Fifth Edition of the Diagnostic and Statistical Manual of Mental Disorders (DSM-5; American Psychiatric Association, 2013), binge eating disorder (BED) is characterised by recurrent binge eating episodes in which a person consumes a large amount of food in a discrete period of time. These episodes of binge eating are further accompanied by a sense of loss of control. BED is the most common ED with a prevalence ranging between 1–4%. Patients with BED have been further found to suffer from a high rate of both mental and somatic comorbidities (Kessler et al., 2013; Keski-Rahkonen and Mustelin, 2016).

Problems in cognitive functioning have been suggested to be core underpinnings for the development and maintenance of BED. Several reviews have demonstrated impaired response inhibition, executive planning, decision making, cognitive flexibility, as well as increased attentional biases and reward sensitivity to food related stimuli among individuals with BED (Kittel et al., 2015; Kessler et al., 2016; Giel et al., 2017b; Stojek et al., 2018). These concepts are all related to the personality trait impulsivity (Dawe and Loxton, 2004; Gullo et al., 2014; Sharma et al., 2014), and among patients with BED this is expressed through impulsive food-related behaviours (Giel et al., 2017b).

Regarding cognitive functions, several inhibitory control tasks (e.g., antisaccade task, Go/No-Go tasks, and Stop Signal task) have been designed to test an individual's ability to stop, change or delay impulsive behavioural responses associated with highly rewarding cues. For instance, in the food-specific antisaccade task which is also administered in this study, participants are asked to look in the opposite direction of the stimulus as quickly as possible when a food-related stimulus appears on the computer screen (Giel et al., 2017a). In such inhibitory control tasks, individiuals with BED experience greater difficulty in suppressing the dominant response, thus demonstrating deficits in inhibitory control towards food stimuli (Hege et al., 2015; Preuss et al., 2019).

In addition to behavioural investigations, event-related potentials (ERPs) derived from electroencephalography (EEG) recordings that measure cortical activity with a high temporal resolution have been used to investigate food-related cognitive processes including response inhibition (Svaldi et al., 2010; Luck, 2014; Leehr et al., 2018; Chami et al., 2019). While making a decision about which ERP components need to be used, characteristics of the stimuli (e.g., sensory, auditory, visual) and targeted cognitive processes are taken into consideration (Luck, 2014). Especially, the inhibitory control related components N2 (observed around 200–300 ms after stimulus presentation), P3 (observed around 300–600 ms after stimulus presentation) and error-related brain potentials (ERN; observed around 50–150 ms after erroneous behaviour) appear to be of particular interest in response inhibition studies. Therefore, for the scope of the current study, we are focusing on N2, P3 and ERN components. The N2 component is a negative deflection that is associated with inhibitory control, conflict monitoring, and automatic response tendencies (Falkenstein, 2006; Leehr et al., 2018; İceta et al., 2020). Particularly N2b (300–360 ms) plays an important role in the attentional detection of deviation from perceptual novelty or deviation from a dominant visual stimulus (Kopp et al., 1996; Folstein and Van Petten, 2008). During the antisaccade task, it is expected that N2 amplitude should be more pronounced, when behaviour is inhibited (correct saccades), in comparison to disinhibited behaviour (erroneous saccades). Another core psychophysiological component that is associated with error processing is the error negativity (Ne; Falkenstein et al., 1991) also known as the error related negativity (ERN; Gehring et al., 1993). ERN/Ne is a sharp negative-going deflection that can be detected after both conscious and unconscious errors (Nieuwenhuis et al., 2001). During the antisaccade task, it is expected that ERN amplitude should be more pronounced during disinhibited behaviour (erroneous saccades), in comparison to inhibited behaviour (correct saccades). The P3 component is a positive deflection that is associated with various functions such as attention, memory, motivation and response inhibition. P3a is generally enhanced within frontocentral electrodes and shown to be relevant to tasks involving inhibition of an overt response (Dimoska et al., 2006; Gajewski and Falkenstein, 2013). Meanwhile, P3b is more enhanced within parietal electrodes and has been shown to be relevant in motivating attention (Chami et al., 2019). More specifically, it is associated with attentional biases towards food given its rewarding nature across different weight and age groups (Nijs et al., 2008; Hill et al., 2013; Hofmann et al., 2015; Biehl et al., 2020; İceta et al., 2020). During the antisaccade task, it is expected that P3 amplitude should be increased during behaviour inhibition (correct saccades) due to increased inhibitory control, or else during behaviour disinhibition (erroneous saccades) due to the attention-grabbing properties of food.

While an extensive number of studies have investigated ERPs related to inhibitory control mechanisms, cross-sectional ERPs studies on response inhibition towards food are limited with heterogeneous findings. One such study by Leehr et al. (2018) examined inhibitory control with a food-related antisaccade task under negative mood conditions in individuals with BED using a combination of eye tracking (ET) and EEG. The authors found significantly larger N2 latencies in overweight individuals without BED than in overweight individuals with BED. ERN/Ne amplitudes were increased for erroneous saccades in comparison to correct saccades regardless of weight or BED status. Meanwhile, through use of the auditory oddball paradigm, İceta et al. (2020) recently found that participants with obesity showed a reduction in P3 and N2 amplitude compared to normal-weight participants, regardless of food disinhibition problems. The authors found that especially the N2 amplitude was associated with clinical markers (i.e., higher self-reported drive for thinness and binge eating), within this group of participants. Another ERP study conducted with a sample of adolescents showed that only those participants with healthy weight had significantly higher P3 amplitudes towards high-calorie food, as opposed to low-calorie food or neutral items. These effects were not found among participants with overweight/obesity (Biehl et al., 2020).

Throughout the literature, it has been emphasised that the inhibition skills and psychopathology of individuals with BED may show improvement following interventions and training programs targeting food-related impulsivity (Giel et al., 2017a; Brockmeyer et al., 2019; İnce et al., 2021). For example, a recent pilot trial of our workgroup was conducted to test the efficacy of a food-modified antisaccade task combined with transcranial direct current stimulation (tDCS) to improve response inhibition in patients with BED (Max et al., 2021). Patients underwent anodal verum and sham tDCS stimulation of the right dorsolateral prefrontal cortex (dlPFC) in a crossover placebo-controlled design. Within three sessions, the error rate and latencies of correct saccades were decreased, indicating improved response inhibition. Although no effect was found regarding the error rate of antisaccades following tDCS administration, receiving 2 milliampere (mA; an indicator of electrical current intensity) stimulation could significantly reduce the latencies of correct saccades compared to sham stimulation. Thus, a response inhibition training for patients with BED seems fruitful. However, it remains unknown whether underlying neurocortical mechanisms related to response inhibition towards food would change as well. To investigate this effect, Lapenta et al. (2014) aimed to reduce food craving in healthy females through increased inhibitory control with tDCS of the dlPFC. In a subsequent Go/No-go task, decreased N2 negativity and significant increase in P3 positivity were found for No-go stimuli following active tDCS. Another study examined the behavioural and ERPs changes following a food-specific Go/No-go task as inhibitory control training in patients with BN and BED in comparison to a control training (Chami et al., 2020). Neither of these interventions significantly changed the N2 or P3 amplitudes from baseline to post-intervention. These heterogenous findings imply that food-specific inhibition trainings might not change ERPs (Chami et al., 2020), but that tDCS could have an effect on ERPs (Lapenta et al., 2014). In the current pilot study, we aim to gain a more in-depth understanding of the potentially underlying neuropsychological mechanisms of inhibitory control in patients with BED. Through this specialised focus on neurophysiological mechanisms, we investigate the EEG activity of a subgroup of patients with BED previously assessed during a study by Max et al. (2021). More specifically, we investigate the following hypotheses:

I. During the food-modified antisaccade task in patients with BED at baseline (T0), there will be differences between erroneous vs. correct saccades for the mean amplitudes of ERPs. In details, we expect the N2 amplitude to be less pronounced in erroneous vs. correct saccades due to decreased response inhibition. Meanwhile, we expect the ERN amplitude to be more pronounced in erroneous vs. correct saccades. Concerning P3 amplitude, we expect that erroneous than correct saccades differ as well, though based on the evidence cited above, the direction is unclear.
II. The three ERP mean amplitudes (N2, ERN, P3) will be associated with the task performance and they will predict behavioural task performance during the T1 and T2 study appointments with verum vs. sham stimulation.
III. The three ERP mean amplitudes (N2, ERN, P3) will be associated with clinical markers at baseline, e.g., eating pathology, trait impulsivity and food-related impulsivity.

## Methods

### Study Design

**Figure 1:**
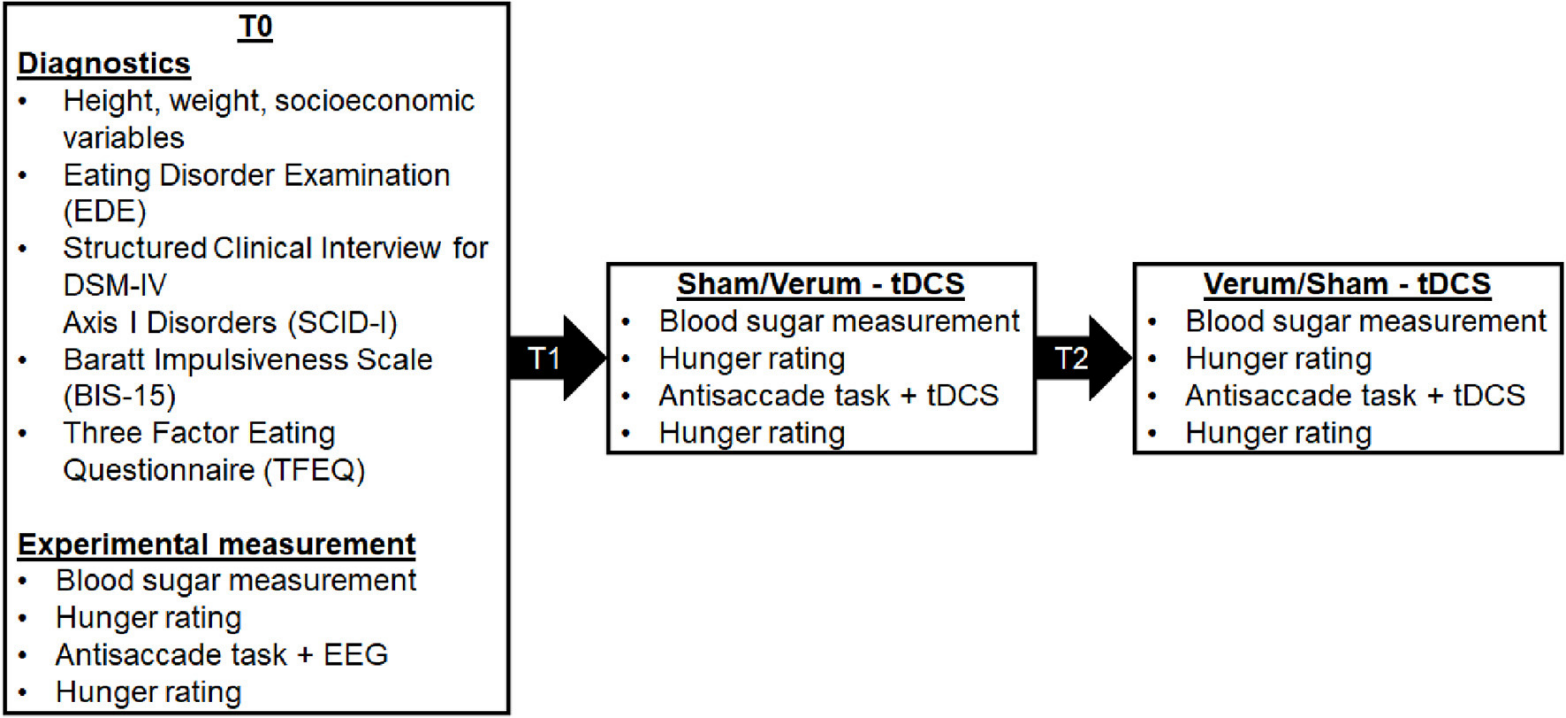
An overview of the study appointments (T0, T1, T2) and the assessed data. The allocation to the two stimulation conditions (verum and sham stimulation) was randomised, counterbalanced and double-blind.

An overview of the study design can be seen in Figure 1. In summary, we completed EEG assessments during baseline measurement (T0) while patients completed the food-modified antisaccade task. We repeated the antisaccade task while patients received tDCS stimulation (verum vs. sham) in a cross-over design at T1 and T2. In the pilot study from Max et al. (2021), we additionally investigated the influence of tDCS and cognitive training on inhibitory control with different stimulation intensities (1 mA vs. 2 mA), however, this is not the focus of the current study.

### Participants

Patients in the current study were a subgroup of the sample recruited by Max et al. (2021). All patients were right-handed adults (age range 20–63) with a diagnosis of BED. Patients with normal weight or overweight/obesity (BMI > 18.5 kg/m^2^) were included. Exclusion criteria included a diagnosis of attention deficit hyperactivity disorder (ADHD), psychotic disorders, bipolar-I disorder, current alcohol or drug addiction, current suicidality, current pregnancy, current physical illnesses which influence weight or eating behaviour, unstable medication (changed medication within the past two weeks), neurological diseases, current prescription of neuroleptics or benzodiazepines, current attendance to structured dieting programs, past bariatric operations, metallic implants in the head and eye diseases.

From a sample of 60 initially interested individuals in the whole project (Max et al., 2021), nine declined interest and 20 were excluded as they did not fulfil inclusion/exclusion criteria (*n* = 8 no BED; *n* = 5 ADHD; *n* = 3 BMI; *n* = 2 bariatric surgery; *n* = 1 seizure; *n* = 1 implausible symptoms). Thus, 31 patients were included in the whole project and completed the assessments. Selection criteria for the subgroup of this study (*N* = 16) are described in detail in the section *data cleaning*.

### Procedure

An overview of the study procedure is shown in Figure 1. Each of the three study appointments were at least one week apart. To control for circadian effects, all patients were invited to complete their assessments at the same time during the late afternoon/evening. To keep homeostatic effects constant, patients were instructed to fast for at least for 4 h prior to their appointments. This was confirmed through analyses of patients' blood sugar levels, as well as hunger levels using a visual analogue scale ranging from 0 cm (not hungry) to 10 cm (very hungry).

At the first study appointment (T0), height, weight and socioeconomic variables were assessed. Two structured clinical interviews for current eating disorders and other psychiatric comorbidities were conducted to control for inclusion and exclusion criteria (EDE, Hilbert et al., 2004; SCID-I, Wittchen et al., 1997). We modified the German Version of the EDE so that an average of one binge eating episode per week over a period of 3 months was necessary to diagnose BED, as in accordance with the DSM-5. To characterise the sample, patients filled out two standardised questionnaires, i.e., the Barrat Impulsiveness Scale (BIS-15; Meule et al., 2011) and the Three-Factor Eating Questionnaire (TFEQ; Pudel and Westenhöfer, 1989). During the T0 appointment, the experimental measurement of the food-modified antisaccade task with concurrent EEG was also conducted. During appointments T1 and T2, verum or sham tDCS was applied while executing the food-modified antisaccade task.

### Questionnaires

#### Barratt Impulsiveness Scale (BIS-15)

Impulsivity as a personality trait was assessed using the BIS-15 (Meule et al., 2011). Three subscales characterise impulsivity: non-planning, motor and attentional impulsivity, while a total score is used as a marker of general impulsivity. A greater degree of impulsivity is indicated by a higher score on the corresponding scale.

#### Eating Disorder Examination (EDE)

The EDE is a semi-structured interview used to assess eating disorders (Hilbert et al., 2004). A total score indicates the severity of the total eating disorder pathology.

#### Three-Factor Eating Questionnaire (TFEQ)

Behavioural, cognitive and affective components of eating behaviour was assessed using the TFEQ (Pudel and Westenhöfer, 1989). Three subscales conceptualise the different facets of eating behaviour: restraint, disinhibition and hunger. The severity of each facet of eating behaviour is indicated by a higher score on the corresponding scale.

### Food-Modified Antisaccade Task

**Figure 2:**
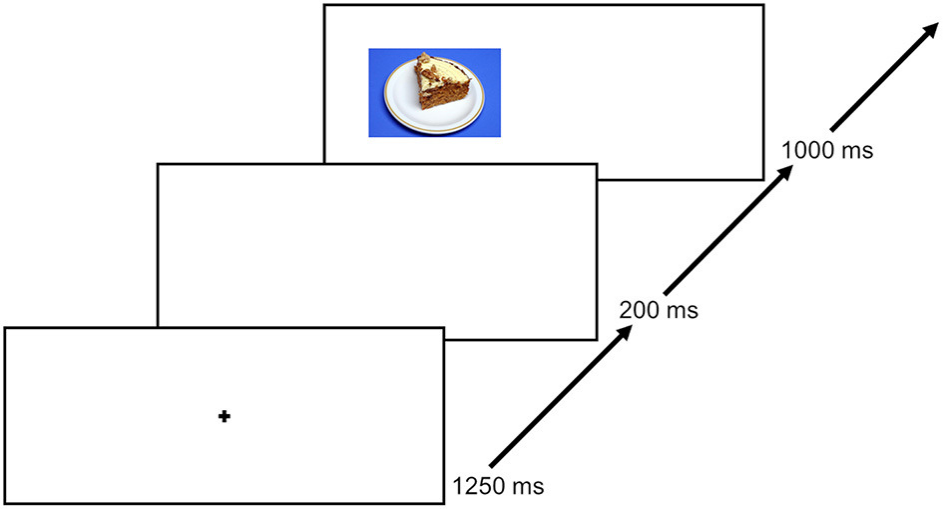
An exemplary trial course. Each trial starts with a 1,250 ms lasting fixation, followed by a 250 ms ISI and thereafter a presentation of a food picture for 1,000 ms. Only food stimuli were used. The next trial starts again with the fixation. The figure was previously published by Max et al. (2021).

This food-modified antisaccade task has been used in numerous studies (e.g., Schag et al., 2013; Leehr et al., 2018). An exemplary trial is shown in Figure 2. Patients in the current study were instructed to look at the fixation cross in the middle of the screen at the beginning of each trial for 1,250 ms. After an interstimulus interval (ISI) of 200 ms, a food picture was shown randomly on the left or right side of the screen for 1,000 ms. Each of the 40 food pictures was presented four times, counterbalanced on the left and right side of the screen throughout the experiment. Patients were instructed to look in the opposite direction of the picture as fast as possible after the food picture appeared on the screen (i.e., they were asked to perform an antisaccade). In total, each patient underwent 160 trials.

### Stimuli and Stimulus Presentation

Forty coloured pictures of high-caloric food (400 x 295 pixels) served as stimulus material. The stimulus material depicted processed sweet or savory foods (e.g., chips, pizza, cookies, burger, chocolate). The stimuli were pre-tested in previous studies addressing response inhibition (Leehr et al., 2016; Giel et al., 2017a). The pictures were presented on a 15.6-inch laptop screen (1280 x 1024 pixels). The food pictures were rated on a visual analogue scale ranging from −5 to 5 concerning palatability (“very unappetizing” to “very appetizing”), wanting to eat the depicted food now (“not at all” to “very”) and liking the food in general (“not at all” to “very”).

### Apparatus

#### Eye Tracking

Eye movements were recorded with SMI RED250mobile (250 Hz sampling rate, 0.4° gaze position accuracy) and iViewRed software. The mobile eye tracker was attached below the laptop screen and was placed 30 cm in front of the patient.

#### Eye Movement Data Processing

Raw data was analysed with BeGaze 3.7 using velocity-based default algorithms that detect fixations and saccades. MatLab R2017b (The Mathworks, Natick, MA, United States) was used for data cleaning and computing output variables, i.e., trial classification (correct vs. erroneous saccade). The error rate (saccades towards the food stimulus) was used as a marker of response inhibition (Hutton and Ettinger, 2006). A trial was excluded if participants did not look at the fixation cross at the beginning of a trial, if there were technical problems or if the saccades started below 80 ms or above 900 ms as these saccades were considered premature/delayed (Schag et al., 2013; Leehr et al., 2018). Single datasets from T0, T1 and T2 (verum or sham condition) with <30 valid eye tracking trials were discarded. The amount of included datasets are described in detail in the section data cleaning.

#### Electroencephalography Recording

The electroencephalogram (EEG) was recorded using an elastic cap (EASYCAP GmbH, Herrsching, Germany), the actiCHamp amplifier system with 32 active Ag/AgCl electrodes and the corresponding Brain Vision Recorder System (Brain Products GmbH, Gilching, Germany). Twenty seven scalp sites (FP1, F7, F3, Fz, F4, F8, FC5, FC1, FCz, FC2, FC6, C3, Cz, C4, CP5, CP1, CPz, CP2, CP6, P7, P3, Pz, P4, P8, O1, Oz, O2) were used to register the EEG. Two electrodes were placed about one centimetre left and right of the eyes for horizontal eye movements, another electrode was placed around one centimetre below the left eye and the FP1 electrode was used to detect vertical eye movements. One electrode was placed on each the left and the right mastoid. The left mastoid was used as an online reference, while the forehead electrode was used as a ground electrode. The online sampling rate was 1,000 Hz and impedances were kept below 10 kΩ before recording.

#### Electroencephalography Data Processing

EEG data was analysed using the MatLab R2017b EEGLAB toolbox (Delorme and Makeig, 2004) and the EEGLAB toolbox ERPLAB (Lopez-Calderon and Luck, 2014). Raw EEG data was resampled offline to 250 Hz and re-referenced to an average of the left and right mastoids. Multiple automated and manual artefact rejection was done (Luck, 2014): Butterworth band-pass filter with a low and high cut-off of 0.1 and 35 Hz and a Notch- filter at 50 Hz were applied. Artefacts were removed using automated independent component analysis (ICA, runica algorithm) (Winkler et al., 2011, 2014). Afterwards, ICA for artefact correction and artefact rejection were conducted manually through visual inspection by the author (Bİ) who was blinded to the experimental conditions. Stimulus locked epochs were extracted ranging from −100 to 1,000 ms, relative to the food picture stimulus. Behaviour locked epochs were extracted ranging from −100 to 500 ms, relative to a correct or erroneous saccade. A baseline correction with 100 ms before stimulus onset or behavioural onset was conducted within the epoched EEG. We decided to use a relatively short baseline to prevent the inclusion of visuomotoric preparation effects (Leehr et al., 2018). For the epoched EEG, artefact correction for the critical channels of the latter-built ERPs (see below) was conducted: Epochs containing EEG signals exceeding an amplitude of 65 μV within a 100 ms time window, or those exceeding −65 to +65 μV within the epoch were considered artefacts and were rejected.

According to the literature, ERPs are built out of three channels (see below). For each ERPs, we analysed mean amplitude, as this method is less noisy and more consistent than peak analyses (Luck, 2014). Time windows for the ERPs were based on visual inspection of ERPs waves, as well as the localisation and time course of the highest ERPs activity over the scalp (Luck, 2014). We excluded all patients with <8 valid ERPs epochs from erroneous or correct trials at T0 (see section data cleaning; Cohen and Polich, 1997; Olvet and Hajcak, 2009; Rietdijk et al., 2014). For the N2 analyses, the stimulus locked epochs were used and consisted of the average of the three frontocentral sites: Cz, Fz, Fcz (Espinet et al., 2012). We determined a time window ranging from 100 to 250 ms after stimulus onset which is in line with previous studies (Leehr et al., 2018; Biehl et al., 2020; Chami et al., 2020). The peaks of the ERP, as well as the highest N2 activity located frontocentral/posterior (Cz), further matched this time window. For the error-related negativity (ERN) behaviour locked epochs were used and consisted of the average of the three frontocentral sites: Cz, Fz, Fcz (Falkenstein et al., 1991). We determined a time window ranging from 50 to 150 ms after the behavioural onset which is in line with previous studies (Nieuwenhuis et al., 2001; Leehr et al., 2018). The highest frontocentral ERN activity for both correct and erroneous saccades were also within this time window. For the P3 analyses, the stimulus locked epochs were used and consisted of the average of the three centro-parietal sites: Cz, CPz, Pz (Sutton et al., 1965; Johnson, 1993; Sommer et al., 2021). We determined a time window ranging from 200 to 400 ms after stimulus onset, as the peaks of the ERPs as well as the highest parietal activity are located within this time window. This is somewhat earlier than what has been found in previous studies (i.e. Lapenta et al., 2014; Biehl et al., 2020; Chami et al., 2020), but nevertheless within the normal range (see Luck, 2014).

#### Transcranial Direct Current Stimulation (tDCS)

Transcranial direct current stimulation (tDCS) was applied by two electrodes (5 x 7 cm) prepared with Ten20 conductive paste (Weaver and Company, Aurora, CO, USA). The electrodes were connected to a battery-driven, constant-current stimulator (DC-STIMULATOR MC, NeuroConn GmbH, Ilmenau, Germany). Placing the anodal electrode over the right dlPFC and the cathodal electrode on the left deltoid muscle, we aimed to increase excitability exclusively of the right dlPFC. The international 10–20 system of electrode placement helped to target the dlPFC by placing the anode over F4 (Jasper, 1958). For the placebo-condition, after the fade-in of 5 s the current was only applied for 46 s, resulting in typical perceived tDCS-sensations (e.g. tingling) and therefore serving as a valid placebo condition (Paulus, 2003).

### Data Cleaning

According to the exclusion criteria of eye tracking and EEG data, *n* = 6 patients had to be excluded from data analysis because of EEG artefact rejection, *n* = 2 because of <30 valid eye tracking trials at T0, *n* = 1 because of <8 valid epochs with correct saccades at T0, *n* = 5 because of <8 valid epochs with erroneous saccades at T0 and *n* = 1 because of corrupted EEG recording. Thus, a total of 16 patients could be included in the final data analyses. Concerning regression analyses, *n* = 1 patient was additionally excluded at T2/sham condition, because of <30 valid eye tracking trials, so that *n* = 15 patients were included in the respective regression analysis. Patients had on average *M* = 100.8 (*SD* = 30.8) valid eye tracking trials at T0, *M* = 51.6 (*SD* = 33.8) ERPs epochs from correct trials, as well as *M* = 49.2 (*SD* = 32.6) valid ERPs epochs from erroneous trials. Patients had *M* = 106.7 (*SD* = 23.3) valid eye tracking trials at T1/verum/sham condition and *M* = 93.1 (*SD* = 37.0) valid eye tracking trials at T2/sham/verum condition.

### Statistical Analysis

All statistical inferences were conducted on a significance level of 95% using SPSS Statistics for Windows (version 24.0). As this is a pilot study, we did not want to inflate beta error propability so that we decided to correct for multiple comparisons only in the case of explorative and multiple testing (see hypothesis 3). To investigate differences in mean amplitudes of erroneous and correct saccades concerning the N2, P3 and ERN during the food-modified antisaccade task (hypothesis 1), paired two-tailed *t*-tests were conducted. Mean amplitudes of ERPs served as dependent variables. All ERPs variables were normally distributed. Sensitivity analyses with stricter cut-offs (N2: >20 trials, P3: >14 trials), other time windows (N2, P3) or single channels (P3 parietal activity) did not lead to deviating results.

Concerning hypothesis 2, we looked at the association between EEG activity and performance in the food-modified antisaccade task, as well as the predictive value of EEG activity on task performance at follow-up appointments (hypothesis 2), a stepwise statistical procedure was used, due in part to the small sample size. We computed correlations by Pearsons's correlation test between the mean amplitude of the ERPs variables (N2, P3 and ERN) in erroneous and correct saccade trials with the performance in the food-modified antisaccade task (mean percentage of erroneous saccades) at baseline and the follow-up appointments that were pooled for the stimulation condition (verum, sham). We further, computed correlations with the verum and sham condition that were pooled for the order of the follow-up appointments (T0, T1, T2). Thereafter we included the variables of the significant correlations stepwise into a regression model, while comparing the model-fits with ANOVA. In the regression analyses, we investigated erroneous saccades as outcome only at the follow-up assessments, not at baseline.

To investigate hypothesis 3, we looked at the association between EEG activity and clinical markers at T0 (eating pathology: EDE total score, binge eating frequency, BMI; trait impulsivity: BIS-15 subscales; food-related impulsivity: TFEQ subscales) by conducting Pearson's correlation tests. All variables were normally distributed besides binge eating frequency in the past four weeks. For the correlations with clinical markers, we Bonferroni-corrected for multiple testing with factor 3 as there were three different clinical markers (eating pathology, trait impulsivity, food-related impulsivity), resulting in a significance level of *p* = 0.0167.

## Results

### Sample Characteristics and Stimulus Ratings

After data cleaning, 16 patients (14 female, two males) were included in the analyses. Further patient characteristics are described in Table 1. Paired two-tailed *t*-tests revealed that the antisaccade task error rate (%) at T1 did not significantly differ from T0 (*t*_(15)_ = 1.60, *p* = 0.131), nor did T1 significantly differ from T2 (*t*_(14)_ = 0.82, *p* = 0.426). The antisaccade task error rate (%) at T2 was significantly lower than at T0, *t*_(14)_ = 2.38, *p* = 0.032. An unpaired two-samples *t*-test revealed no significant difference between sham and verum stimulation, *t*_(9.89)_ = −0.31, *p* = 0.762. Concerning food valences, the stimuli were rated overall positively (*M*_palatability_ = 1.60, *SD*_palatability_ = 2.56; *M*_wanting_ = 1.22, *SD*_wanting_ = 3.24; *M*_liking_ = 1.93, *SD*_liking_ = 2.96).

**Table 1:** Sample characteristics at baseline.

	**N**	**M**	**SD**
Age	16	38.6	13.6
BMI (kg/m^2^)	16	33.7	10.9
binge eating episodes in the past 4 weeks acc. to EDE	16	15.6	13.4
EDE total score	16	2.0	.9
BIS-15 non-planning subscale	16	10.5	2.3
BIS-15 motor subscale	16	10.4	2.1
BIS-15 attentional subscale	16	8.5	2.5
TFEQ restraint	16	6.3	3.5
TFEQ disinhibition	16	11.3	3.2
TFEQ feeling hungry	16	10.3	2.5
Antisaccade task error rate (%) T0	16	47.6	26.2
Antisaccade task error rate (%) T1	16	40.5	30.4
Antisaccade task error rate (%) T2	15	40.0	26.2

*BIS-15, baratt impulsiveness scale; EDE, eating disorder examination; TFEQ, three-factor eating questionnaire*.

### Mean Amplitude of ERPs in Erroneous vs. Correct Saccades at Baseline

**Figure 3:**
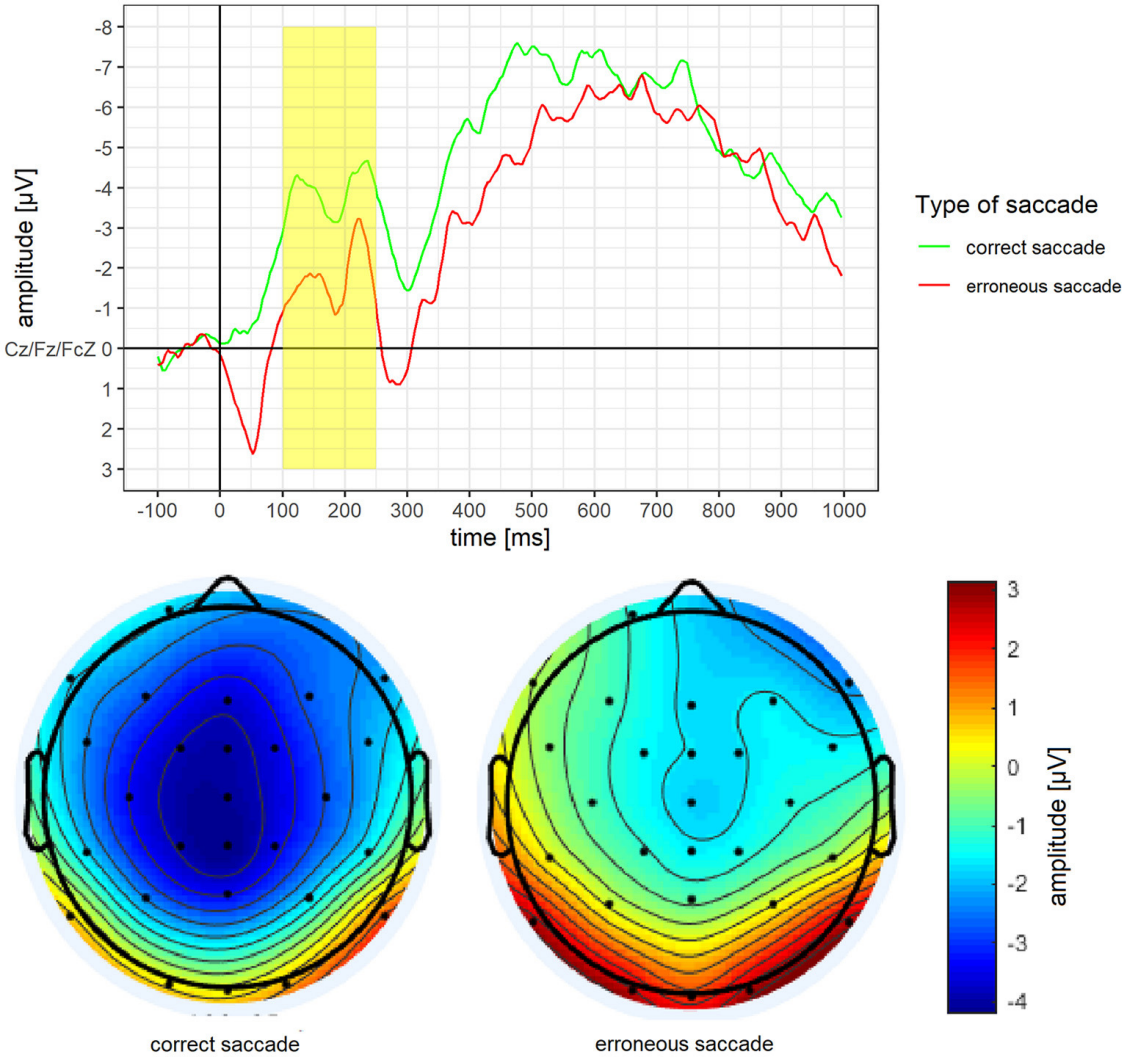
Grand average difference wave of the N2 separately for erroneous and correct saccades. Underneath a scalp map displaying the mean voltage distribution for erroneous and correct saccades in the time window ranging from 100 to 250 ms after stimulus onset.

Mean amplitude and mean activity over the scalp for the N2 of erroneous and correct saccades are depicted in Figure 3. For the N2, a significantly less pronounced mean amplitude for erroneous saccades (*M* = −1.74 μV, *SD* = 3.32) than for correct saccades (*M* = −3.90 μV, *SD* = 3.45) was observed, *t*_(15)_ = 3.46, *p* = 0.004, *d* = 0.86.

**Figure 4:**
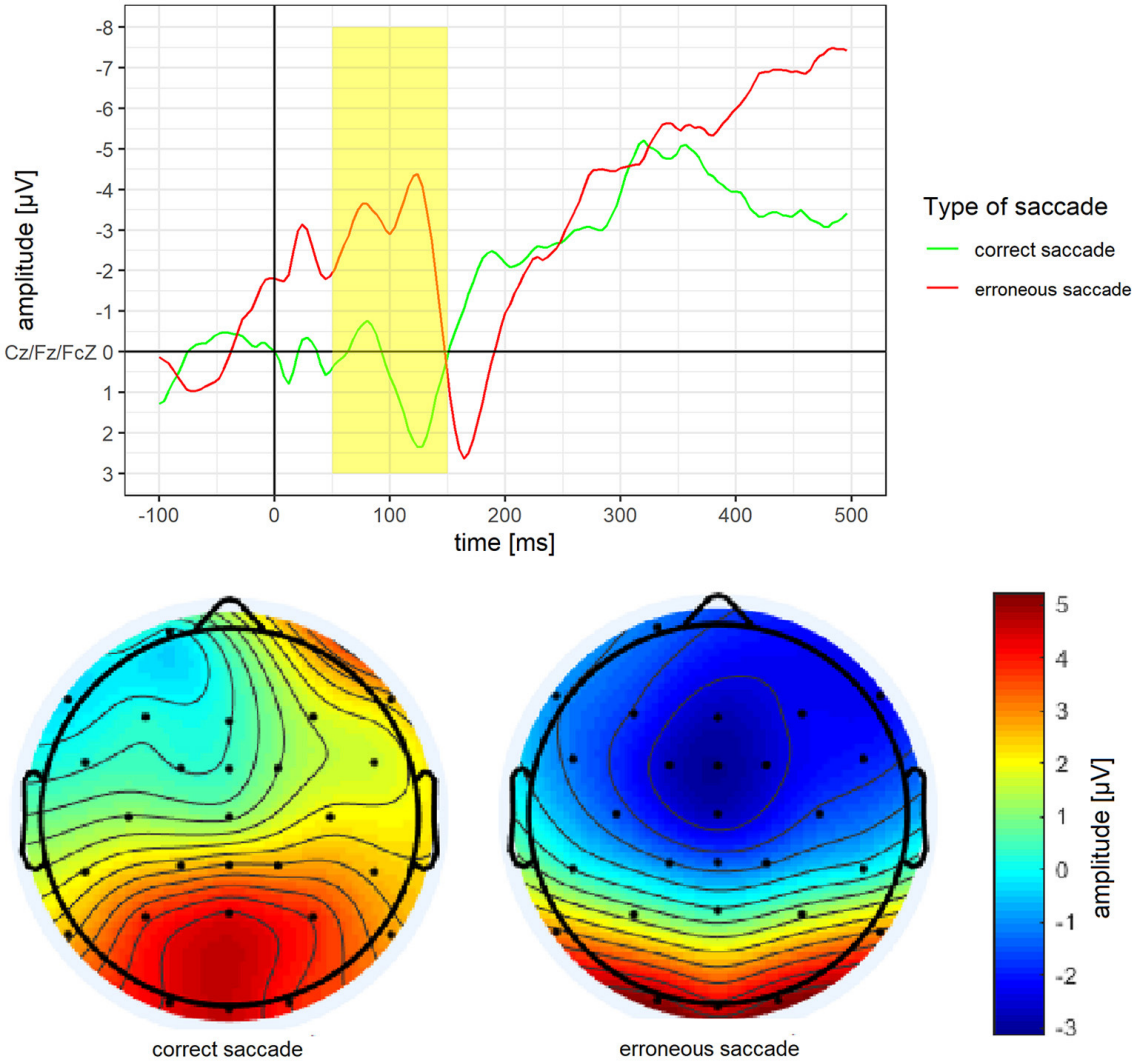
Grand average difference wave of the ERN separately for erroneous and correct saccades. Underneath a scalp map displaying the mean voltage distribution for erroneous and correct saccades in the time window ranging from 50 to 150 ms after saccade onset.

Mean amplitude and mean activity over the scalp for the ERN of erroneous vs. correct saccades are depicted in Figure 4. For the ERN, a significantly more pronounced mean amplitude for erroneous saccades (*M* = −2.87 μV, *SD* = 3.02) than for correct saccades (*M* = 0.62 μV, *SD* = 3.87) was observed, *t*_(15)_ = −3.37, *p* = 0.004, *d* = 0.84.

**Figure 5:**
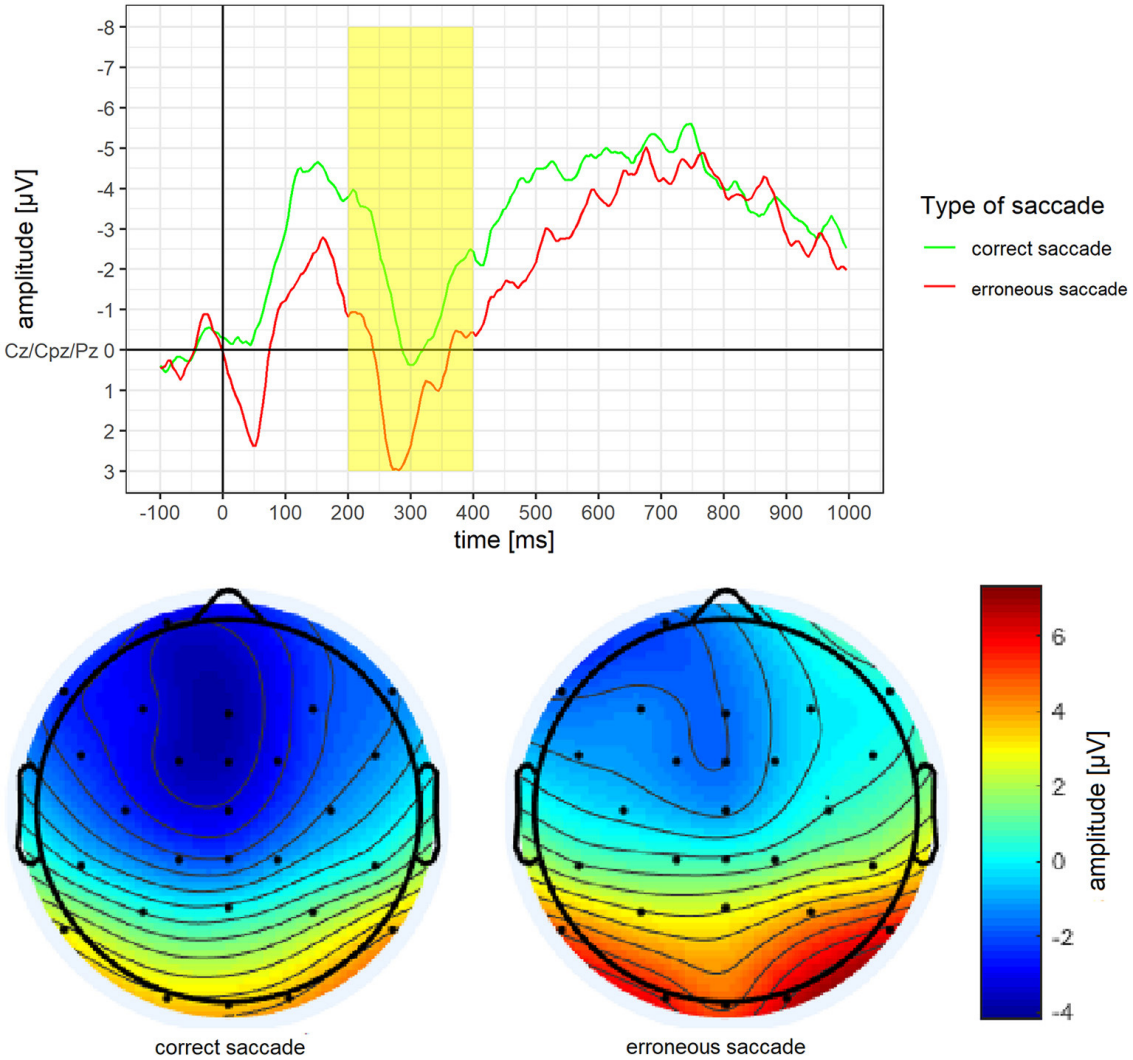
Grand average difference wave of the P3 separately for erroneous and correct saccades. Underneath a scalp map displaying the mean voltage distribution for erroneous and correct saccades in the time window ranging from 200 to 400 ms after stimulus onset.

Mean amplitude and mean activity over the scalp for the P3 of erroneous vs. correct saccades are depicted in Figure 5. For the P3, a significantly more pronounced mean amplitude for erroneous saccades (*M* = 0.77 μV, *SD* = 2.63) than for correct saccades (*M* = −1.66 μV, *SD* = 4.22) was observed, *t*_(15)_ = 3.14, *p* =.007, *d* = 0.79.

### Associations Between ERPs and Performance in the Food-Modified Antisaccade Task at Baseline and Follow-Up Appointments

The correlations between ERPs and performance in the food-modified antisaccade task are shown in Supplementary Table 1. There were neither significant correlations between the N2 and the mean percentage of erroneous saccades nor between the ERN and the mean percentage of erroneous saccades. However, there were significant correlations between the mean amplitude of the P3 in erroneous saccades and the mean percentage of erroneous saccades at T0 (*r* = −0.64, *p* = 0.007), T1 (*r* = −0.65, *p* = 0.007), T2 (*r* = −0.64, *p* = 0.010), under verum stimulation (*r* = −0.67, *p* = 0.005) and under sham stimulation (*r* = −0.62, *p* = 0.015). Furthermore, there were significant correlations between the mean amplitude of the P3 in correct saccades and the mean percentage of erroneous saccades at T0 (*r* = −0.70, *p* = 0.002), at T1 (*r* = −0.52, *p* = 0.039) and under verum stimulation (*r* = −0.57, *p* = 0.022), but no significant correlation with sham stimulation (*r* = −0.43, *p* = 0.109).

These significant correlations with the follow-up assessments were entered stepwise into linear regression analyses. The mean amplitude of the P3 in erroneous saccades significantly predicted the mean percentage of erroneous saccades at T1 (β = −7.44, *SE* = 2.36, *p* =.007), at T2 (β = −6.23, *SE* = 2.06, *p* = 0.010), under verum stimulation (β = −7.22, *SE* = 2.14, *p* = 0.005) and under sham stimulation (β = −6.47, *SE* = 2.30, *p* = 0.015).

### Association Between ERPs and Clinical Markers at Baseline

All correlations between ERPs and clinical markers at baseline are presented in Supplementary Table 2. No significant correlations were observed between ERPs and eating disorder pathology (i.e., binge eating frequency, EDE scores, BMI). However, ERPs did partially correlate with trait impulsivity. The BIS-15 non-planning subscale showed a significant correlation with the mean amplitude of the N2 of correct saccades (*r* = −0.59, *p* = 0.016), the mean amplitude of the ERN of correct saccades (*r* = −0.54, *p* = 0.032) and the mean amplitude of the P3 of correct saccades (*r* = −0.59, *p* = 0.017). However, after Bonferroni-correction, only the association between the BIS-15 non-planning subscale and the N2 remained significant (*p* <0.0167). While the N2 and ERN did not correlate with food-related impulsivity, a significant correlation could be found between the mean amplitude of the P3 in erroneous saccades and the TFEQ subscale restraint (*r* = −0.61, *p* = 0.012) as well as the subscale disinhibition (*r* = −0.67, *p* = 0.004).

## Discussion

This pilot study aimed to investigate the underlying neuropsychological mechanisms of an inhibitory control training combined with tDCS by analysing the ERPs (i.e., N2, ERN and P3) of patients with BED while executing the food-modified antisaccade task. Concerning hypothesis 1, we found significant differences between erroneous and correct saccades for the mean amplitudes of the ERPs. As expected, N2 mean amplitude was less pronounced in erroneous saccades vs. correct saccades, while ERN and P3 mean amplitudes were more pronounced in erroneous saccades vs. correct saccades. Concerning hypothesis 2, baseline P3 predicted the performance in the food-specific antisaccade task during verum and sham stimulation of the right dlPFC through tDCS at follow-up appointments. In terms of clinical markers (hypothesis 3), we demonstrated a significant association between N2 mean amplitude in correct saccades with non-planning behaviour of BIS-15, and between P3 mean amplitude in erroneous saccades with restraint and disinhibition of TFEQ.

Taking a closer look at our first hypothesis, N2 mean amplitude was significantly less pronounced in erroneous saccades vs. correct saccades indicating enhanced inhibition during correct saccades compared to erroneous saccades. This finding is consistent with previous research demonstrating increased N2 mean amplitudes in the case of increased cognitive conflict and difficulty in response inhibition (Chen et al., 2018). The ERN mean amplitude was significantly more pronounced in erroneous saccades vs. correct saccades with negative activity in erroneous saccades and positive activity in the correct saccades. This is in line with an earlier study combining EEG and ET (Leehr et al., 2018). After the ERN time window (around 150 to 200 ms), the peak from the erroneous trials changes and becomes as positive as the peak from the correct saccades. At this time, inhibition may be particularly pronounced, possibly during or else after correction of the error. This phenomenon could be explained by error positivity (Pe), which has been suggested to be responsible for error recognition and modification of response (Falkenstein et al., 2000; Nieuwenhuis et al., 2001). Thus, after an initially erroneous saccade, the behaviour might be corrected. These places demand on the neural capacities, demonstrated by an elevated ERN amplitude. Unfortunately, we were unable to investigate this hypothesis or further analyse error correction within our data, due to the small sample and frequency of trials. Meanwhile, P3 mean amplitude was significantly more pronounced in erroneous saccades than correct saccades. As previous research has demonstrated enhanced P3b amplitudes towards attentional processing of salient stimuli, i.e., food vs. neutral stimuli (e.g., Nijs et al., 2008; Hill et al., 2013), it is possible that our study included assessments of the P3b component. Although the task required patients to look away from food stimuli, due to the rewarding and attention-grabbing properties of food (see Chami et al., 2019; Biehl et al., 2020), the effects of which are particularly amplified for patients with BED (e.g., Schag et al., 2013), attentional resources were more demanded in erroneous trials, when the patients looked at the food stimuli, i.e., were exposed to food.

Concerning our second hypothesis, task performance did not correlate with N2 or ERN mean amplitudes. This is in line with a previous study showing no effect of food-specific inhibitory control training on N2 amplitude (Carbine et al., 2021). However, the percentage of antisaccade errors in this study was highly correlated with the P3 mean amplitude of erroneous saccade trials, as well as partially correlated with correct saccade trials during T0, T1, T2 or during verum and sham stimulation. These findings suggest that if P3 mean amplitude is more pronounced at baseline assessment, the error rate in the inhibition task will be lower at the measurement points, independent from stimulation (verum or sham). While the comparisons between erroneous and correct saccades in hypothesis 1 were found for all patients, the correlations pertaining to hypothesis 2 were directly related to individual task-relevant performance with P3 activity. Thus, it may be possible that in assessing the P3, we assessed the attention-grabbing properties of food as well as inhibitory control mechanisms. Moreover, the regression analyses revealed that the mean amplitude of the P3 in erroneous saccades significantly predicted the error rates at T1 and at T2 under both, verum and sham stimulation. Thus, P3 could be interpreted as a predictor of the overall task performance as it predicts all measurement points and all stimulation conditions (see also results from Lapenta et al., 2014). This might imply that those who are already able to recruit resources for inhibitory control at baseline, might benefit more from such a training programme. This emphasises the neuromodulatory perspectives tDCS might offer in terms of facilitating inhibitory control. However, it is not possible to discriminate whether this effect was achieved due to training effects from the antisaccade task, tDCS stimulation or a combination of both. Given that P3 significantly predicted performance on both verum and sham conditions, it is more likely that the effect was independent of tDCS stimulation. In this regard, the efficacy of tDCS should be interpreted cautiously.

Our exploratory analysis concerning the third hypothesis, namely whether ERPs are associated with clinical markers of BED did not demonstrate a significant correlation between ERPs and frequency of binge eating episodes in the past four weeks, EDE total score or BMI. This is in accordance with previous research showing no significant association between P3 amplitude and eating psychopathology (Schaefer and Nooner, 2018; İceta et al., 2020). After Bonferroni correction, only N2 mean amplitudes in trials with correct saccades was negatively associated with non-planning behaviour, one facet of trait impulsivity (BIS-15). This is in line with the observation that the correlation between self-reported impulsivity and aspects of impulsive behaviour in a laboratory setting is rather low (Sharma et al., 2014). The strongest correlations between ERPs and self-reports within our study were found between P3 in erroneous saccade trials and food-related impulsivity (TFEQ), in particular with restraint and disinhibition subscales. These findings are further in line with those reported by Schag et al. (2021) who found significant correlations between the antisaccade task and food-related impulsive behaviour, but not with general eating pathology and trait impulsivity. Thus, a higher P3 activity in erroneous saccade trials is associated with less restraint, i.e., less cognitive control and thus more impulsive behaviour towards food. Surprisingly however, higher P3 activity is related as well to less disinhibition, i.e., less food-related impulsivity. This could be because all patients rated very high on this subscale with a mean of 11.5 (*SD* = 3.2), whereas a representative study from Löffler et al. (2015) reports a mean of 4.8 (*SD* = 3.1) for 40–50 year old females of the general population. Thus, within this patient group with very high disinhibition scores, those with less disinhibition had higher P3 activity while executing errors. A concern that has been raised by prior studies is that behavioural tasks and self-report improvements do not actually measure a single impulsivity frame (Sharma et al., 2014; Strickland and Johnson, 2020) and that objective measurements such as ERPs may be more accurate for testing inhibitory control towards food (Carbine et al., 2017). Overall, P3 might be closest to inhibitory control performance in the antisaccade task as it is a predictor for task performance at several study appointments. However, there is need for further investigating the role of P3 as potential marker for food-related inhibitory control processes.

### Strengths and Limitations

Due to the low sample size and a considerable proportion of excluded patients and trials to increase data quality, the reported results are only preliminary and should be interpreted with caution. For instance, it could not be determined if stimulation intensity (1 mA vs. 2 mA) influenced the results. However, the error rate in the antisaccade task did not differ between 1 mA and 2 mA in the verum condition, thereby suggesting an independence of effects from stimulation. Another point is that P3 was assessed at centro-parietal sites, whereas a more frontal dlPFC area was stimulated with tDCS that is related with inhibitory control. This might explain why we observed not only inhibitory control, but also attention motivation processes with P3, while also explaining why P3 predicted task performance independently of verum vs. sham stimulation. Lastly, Barton et al. (2006) have argued that inhibitory processes are different for antisaccade and Go/No-go tasks. In this regard, the psychophysiological constructs that we assessed in this study might be different from previous studies that administered different tasks (e.g., Biehl et al., 2020; Chami et al., 2020; İceta et al., 2020), so that the results cannot be compared directly.

This study also contained several strengths. To the best of our knowledge, this pilot study is one of the rare studies investigating the psychophysiological processes of individuals with BED during a food-specific response inhibition task. A remarkable strength is our investigation of the effect of neurostimulation with a combination of psychophysiological measures. Although studies on tDCS providing evidence for reducing food craving and binge eating behaviour are scarce, studies examining the effects of tDCS along with psychophysiological measures are virtually non-existent. A further strength of our study is the combination of psychophysiological measurement, behavioural task, and self-report instruments in the data collection. Furthermore, rather than relying on a self-report instrument for identifying individuals with BED, two structured clinical interviews were administered during the study.

### Conclusions and Future Directions

This pilot study provides preliminary evidence for differing response inhibition processes among patients with BED when confronted with food through findings of less pronounced N2 and more pronounced ERN and P3 amplitudes in erroneous vs. correct saccades. As it predicts task performance on follow up assessments, P3 might be a potential marker for food-related inhibitory control processes in BED. As P3 predicted performance in the tDCS verum and sham conditions, there is no strong evidence based on this pilot study, that tDCS is a beneficial training adjunct in patients with BED. It might be that the response inhibition training itself might be solely benefial. However, based on previous literature suggesting that active tDCS can be helpful for reducing eating psychopathology (e.g., Burgess et al., 2016; Ljubisavljevic et al., 2016), a combined training consisting of the antisaccade task and anodal tDCS on the right dlPFC might target inhibitory control regions. To further investigate this question, if a combined training is more beneficial than the training task solely, we are currently running a randomised controlled trial (https://clinicaltrials.gov/ct2/show/study/NCT04572087) to enhance cognitive control over eating in patients with BED through six training sessions of anodal tDCS to the right dlPFC in combination with the food-related antisaccade task. Such a training might change underlying inhibitory control mechanisms of binge eating behaviour. For instance, those patients who are able to activate P3 areas from beginning on might benefit more from the training.

Another important point for the future are more neuromodulation studies on food-related impulsivity. Current studies in this field are providing promising findings regarding improve food intake, food craving, binge eating and response inhibition. Nevertheless, studies in patients with BED are too scarce to draw conclusion about their efficacy, and randomised controlled trial with this population are virtually non-existent (İnce et al., 2021). Moreover, the underlying mechanisms of neuromodulation are still not discovered and research concerning this topic is still in its infancy. Although our findings are encouraging, the results of the present pilot study nevertheless will need to be replicated with larger samples and with solutions to our previously described methodological challenges. Thus, we hope that our randomised controlled trial that is based on this project might be an initial step to elucidate the psychophysiological underpinnings of neurostimulation in patients with BED.

Concerning EEG research, previous literature has suggested that alternative interpretations, e.g., attentional bias to rewarding food stimuli, may also be possible. Further research is needed on this subject, given the preliminary nature of the data that is currently available. One such explanation pertains to late positive potentials as an interesting indicator of motivated attention towards salient stimuli (Svaldi et al., 2010; Carbine et al., 2020), which could enrich our understanding of electrophysiological phases of food cue processing. Based on previous research findings (e.g., Nijs et al., 2010; Nikendei et al., 2012; Seo and Lee, 2021), future research might also benefit from investigating whether homoeostasis or shape and weight concern modify electrophysiological and behavioural response inhibition among individuals with BED.

Taken together, our pilot study delivers first insights into the psychophysiological processes of patients with BED while executing a response inhibition task. Our results will engage further research concerning underlying mechanisms and potential interventions in patients with BED.

## Data Availability Statement

The raw data supporting the conclusions of this article will be made available by the authors, without undue reservation.

## Ethics Statement

The study was approved by the Ethics Committee of the Medical Faculty Tübingen, Germany (Project No. 459/2016BO2). The patients/participants provided their written informed consent to participate in this study.

## Author Contributions

KG, CP, KS, and SZ contributed to the study conception and design. Material preparation, data collection, and analysis were performed by SM with support from KS. EL provided the procedure and programme codes for the recording, cleaning and aggregation of EEG data and gave valuable support concerning EEG data. EEG processing was done by SM with support from Bİ and KS. The first draft of the manuscript was written by Bİ, SM, and KS and all authors commented on previous versions of the manuscript. All authors read and approved the final manuscript.

## Funding

This study was funded by a grant from the German Research Council (GI 878/4-1, PL 525/7-1). KS is supported by the Margarete von Wrangell Programme funded by the Ministry for Science and Education Baden-Württemberg, Germany. We acknowledge support by Open Access Publishing Fund of University of Tübingen.

## Conflict of Interest

The authors declare that the research was conducted in the absence of any commercial or financial relationships that could be construed as a potential conflict of interest.

## Publisher's Note

All claims expressed in this article are solely those of the authors and do not necessarily represent those of their affiliated organizations, or those of the publisher, the editors and the reviewers. Any product that may be evaluated in this article, or claim that may be made by its manufacturer, is not guaranteed or endorsed by the publisher.
