# Supplementary material for: A Pilot Event-Related Potentials Study on Mechanisms Underlying a tDCS-Enhanced Food-Specific Response Inhibition Task for Patients With Binge Eating Disorder
Source: Front Psychol. 2021 Oct 12;12:721672. doi: 10.3389/fpsyg.2021.721672 (PMC8546297; doi:10.3389/fpsyg.2021.721672)
Supplement: Supplementary file 1 [file Table_1.docx]

| ERN correct saccade | *r* = -.29,  *p* = .282 | *r* = -.01,  *p* = .973 | *r* = -.05,  *p* = .871 | *r* = -.06,  *p* = .828 | *r* = .01,  *p* = .971 |
| --- | --- | --- | --- | --- | --- |
| ERN erroneous saccade | *r* = -.23,  *p* = .387 | *r* = -.48,  *P* = .062 | *r* = -.35  *p* = .206 | *r* = -.39,  *p* = .133 | *r* = -.44,  *p* = .097 |
| P3 correct saccade | *r* = -.70**,  *p* = .002 | *r* = -.52*,  *p* = .039 | *r* = -.48,  *p* = .068 | *r* = -.57*,  *p* = .022 | *r* = -.43,  *p* = .109 |
| P3 erroneous saccade | *r* = -.64**,  *p* = .007 | *r* = -.65**,  *p* = .007 | *r* = -.64*,  *p* = .010 | *r* = -.67**,  *p* = .005 | *r* = -.62*,  *p* = .015 |
| N2 correct saccade | *r* = -.37,  *p* = .155 | *r* = -.50,  *p* = .050 | *r* = -.26,  *p*= .350 | *r* = -.49,  *p* = .057 | *r* = -.29,  *p* = .295 |
| N2 erroneous saccade | *r* = -.16,  *p* = .549 | *r* = -.29,  *p* = .270 | *r* = .02,  *p* = .941 | *r* = -.29,  *p* = .279 | *r* = -.01,  *p* = .981 |
|  | % erroneous saccades T0 | % erroneous saccades T1 | % erroneous saccades T2 | % erroneous saccades verum stimulation | % erroneous saccades sham stimulation |

**Supplementary Table 1.** Correlations between mean amplitude of the ERP variables and performance in the food-modified antisaccade task.

*Note*. * indicates *p* < .05, ** indicates *p* < .01
