# Supplementary material for: A Pilot Event-Related Potentials Study on Mechanisms Underlying a tDCS-Enhanced Food-Specific Response Inhibition Task for Patients With Binge Eating Disorder
Source: Front Psychol. 2021 Oct 12;12:721672. doi: 10.3389/fpsyg.2021.721672 (PMC8546297; doi:10.3389/fpsyg.2021.721672)
Supplement: Supplementary file 2 [file Table_2.docx]

| ERN correct saccade | *r* = -.54*,  *p* = .032 | *r* = -.25,  *p* = .352 | *r* = -.07,  *p* = .811 | *r* = -.14,  *p* = .611 | *r* = .16,  *p* = .564 | *r* = -.15,  *p* = .568 | *r* = -.29,  *p* = .285 | *r* = -.33,  *p* = .206 | *r* = .18,  *p* = .513 |
| --- | --- | --- | --- | --- | --- | --- | --- | --- | --- |
| ERN erroneous saccade | *r* = -.24,  *p* = .362 | *r* = -.37,  *P* = .154 | *r* = .08  *p* = .756 | *r* = -.39,  *p* = .132 | *r* = -.20,  *p* = .449 | *r* = .02,  *p* = .954 | *r* = .18,  *p* = .514 | *r* = -.04,  *p* = .882 | *r* = .14,  *p* = .610 |
| P3 correct saccade | *r* = -.59*,  *p* = .017 | *r* = -.31,  *p* = .244 | *r* = -.39,  *p* = .136 | *r* = -.45,  *p* = .077 | *r* = -.30,  *p* = .253 | *r* = -.02,  *p* = .946 | *r* = -.28,  *p* = .290 | *r* = .32,  *p* = .225 | *r* = -.15,  *p* = .575 |
| P3 erroneous saccade | *r* = -.12,  *p* = .665 | *r* = .07,  *p* = .785 | *r* = -.15,  *p* = .574 | *r* = **-.61***,  *p* = .012 | *r* = **-.67****,  *p* = .004 | *r* = -.27,  *p* = .318 | *r* = -.08,  *p* = .774 | *r* = .40,  *p* = .124 | *r* = -.27,  *p* = .308 |
| N2 correct saccade | *r* = **-.59***,  *p* = .016 | *r* = -.35,  *p* = .179 | *r* = -.31,  *p*= .251 | *r* = -.25,  *p* = .344 | *r* = -.28,  *p* = .299 | *r* = -.04,  *p* = .872 | *r* = .15,  *p* = .571 | *r* = .22,  *p* = .412 | *r* = -.07,  *p* = .806 |
| N2 erroneous saccade | *r* = -.16,  *p* = .556 | *r* = -.12,  *p* = .654 | *r* = -.08,  *p* = .765 | *r* = -.13,  *p* = .636 | *r* = -.49,  *p* = .054 | *r* = -.06,  *p* = .818 | *r* = -.04,  *p* = .874 | *r* = .22,  *p* = .424 | *r* = -.36,  *p* = .175 |
|  | BIS-15 nonplanning impulsivity | BIS-15 motor impulsivity | BIS-15 attentional impulsivity | TFEQ restraint | TFEQ disinhibition | TFEQ hunger | BMI | Number of self-reported binge eating episodes during the last 7 days | EDE total score |

**Supplementary Table 2.** Correlations between mean amplitude of the ERP variables and clinical markers.

*Note*. *BIS-15* Baratt Impulsiveness Scale; *EDE* eating disorder examination; *TFEQ* three-factor eating questionnaire. * indicates *p* < .05, ** indicates *p* < .01, **bold** indicates significance after Bonferroni-correction (*p* < .0167)
